# Influence of the environment on ragweed pollen and their sensitizing capacity in a mouse model of allergic lung inflammation

**DOI:** 10.3389/falgy.2022.854038

**Published:** 2022-08-05

**Authors:** Shu-Hua Liu, Sahar Kazemi, Gerhard Karrer, Anke Bellaire, Wolfram Weckwerth, Jakob Damkjaer, Oskar Hoffmann, Michelle M. Epstein

**Affiliations:** ^1^Laboratory of Experimental Allergy, Department of Dermatology, Medical University of Vienna, Vienna, Austria; ^2^Institute of Botany, University of Natural Resources and Life Sciences, Vienna, Austria; ^3^Department of Botany and Biodiversity Research, University of Vienna, Vienna, Austria; ^4^Department of Functional and Evolutionary Ecology, Molecular Systems Biology, University of Vienna, Vienna, Austria; ^5^Vienna Metabolomics Center (VIME), University of Vienna, Vienna, Austria; ^6^ALK-Abelló A/S, Hørsholm, Denmark; ^7^Division of Pharmacology & Toxicology, Department of Pharmaceutical Sciences, University of Vienna, Vienna, Austria

**Keywords:** *Ambrosia artemisiifolia*, ragweed pollen, allergy, asthma, mice, environment, climate change

## Abstract

Common ragweed (*Ambrosia artemisiifolia*) is an invasive plant with allergenic pollen. Due to environmental changes, ragweed pollen (RWP) airborne concentrations are predicted to quadruple in Europe by 2050 and more than double allergic sensitization of Europeans by 2060. We developed an experimental RWP model of allergy in BALB/c mice to evaluate how the number of RWP and how RWP collected from different geographical environments influence disease. We administered RWP six times over 3 weeks intranasally to the mice and then evaluated disease parameters 72 h later or allowed the mice to recover for at least 90 days before rechallenging them with RWP to elicit a disease relapse. Doses over 300 pollen grains induced lung eosinophilia. Higher doses of 3,000 and 30,000 pollen grains increased both eosinophils and neutrophils and induced disease relapses. RWP harvested from diverse geographical regions induced a spectrum of allergic lung disease from mild inflammation to moderate eosinophilic and severe mixed eosinophilic-neutrophilic lung infiltrates. After a recovery period, mice rechallenged with pollen developed a robust disease relapse. We found no correlation between Amb a 1 content, the major immunodominant allergen, endotoxin content, or RWP structure with disease severity. These results demonstrate that there is an environmental impact on RWP with clinical consequences that may underlie the increasing sensitization rates and the severity of pollen-induced disease exacerbation in patients. The multitude of diverse environmental factors governing distinctive patterns of disease induced by RWP remains unclear. Further studies are necessary to elucidate how the environment influences the complex interaction between RWP and human health.

## Introduction

*Ambrosia artemisiifolia* (short ragweed) releases pollen that causes allergic rhinoconjunctivitis and asthma. Allergic disease induced by RWP is on the rise in Europe because of a significant increase in the number of plants and the release of high concentrations of pollen. The underlying cause appears to be related to climate change. For example, higher temperatures, changes in precipitation, atmospheric gases like CO_2_ and NO_2_ and air pollution prolong the growing season, increase the spread of the plant, and the number of pollen grains in the air. Computer models predict a quadrupling of pollen concentration in Europe by 2050 and the spreading of plants and pollen to previously unaffected countries like Scandinavia and the UK (1–7). Based on climate change associated increases in airborne pollen, experts predict that the number of ragweed-sensitized Europeans will more than double from 33 to 77 million by 2060 (8).

Several studies have followed clinical allergies related to RWP. One pan-European study showed that ragweed sensitization rates were about 10% across Europe and up to 58% in Hungary (9), with a clear correlation between RWP levels, symptoms of rhinitis or asthma, medication use, and medical consultations (10–13). One study reported a particularly strong association between sensitization in children and RWP concentration at levels higher than 5,000 RWP grains m^−3^ year^−1^ (14). The concentration of airborne pollen correlates with allergic sensitization and disease severity but may also relate to environmental changes that influence the quality of the pollen. In other words, could the environment make the pollen more allergenic? To address this question, we created an experimental RWP mouse model of allergic lung inflammation to test increasing doses of RWP and pollen from diverse geographical and climatic environments to evaluate allergen sensitization and disease severity.

## Materials and methods

### Animals

Female BALB/c mice (6–8-week old) purchased from Charles River Laboratories Inc. (Sulzfeld, Germany) were housed in a specific pathogen-free facility at the Medical University of Vienna in a temperature-controlled environment with a 12-h dark/light cycle and water and food *ad libitum*. All animal experiments were done according to the Animal Care Committee of the Medical University of Vienna and approved by the Austrian Ministry of Education, Science and Research.

### Ragweed pollen

RWP were generously provided by Allergon AB, Ängelholm, Sweden (AG1, AG2), ALK-Abelló, Hørsholm, Denmark (ALK1, ALK2) and were collected by our group in two sites in Austria (VA1, VA2). For details about the pollen samples used in these experiments see Supplementary Table 1. We calculated the number of pollen grains per sample by weight based on a previous study demonstrating roughly 300 RWP grains per μg (15). All pollen were stored at −20°C until use.

### Amb a 1 levels

One dried sample of each RWP underwent extraction in ammonium bicarbonate (NH_4_HCO_3_, VWR Chemicals—Søborg, Denmark), using 125 mM buffer at pH 8.3. The ratio of pollen to the buffer was 4.0 grams pollen to 23 ml of buffer. The extraction was performed for 2 h with continuous stirring with the pH adjusted for ~10, 30 and 60 min. The pollen was then spun down, and the supernatant was filtered through a 0.22 μm filter. The samples then underwent radial immunodiffusion (16). Briefly, agarose gel 1% in TRIS-Veronal buffer (Litex HSA 1000 Agarose) (Lonza—Basel, Switzerland; TRIS from Merck—Barbital (Veronal) Sigma-Aldrich—St. Louis, Missouri, USA) was heated under stirring and afterwards kept in a water bath at ~56°C for at least 15 min before use. The agarose gel was combined with the appropriate volume of a monospecific rabbit polyclonal Amb a 1 antibody (ALK, in-house antibody production), mixed, and then cast on a glass plate. When the gel had solidified, wells were punched at appropriate distances. An ALK in-house reference sample (IHR) in 4 concentrations, and the pollen extract samples, were applied to the wells, and the gel was placed in a humidity chamber (to prevent the gel from drying out) for 48–72 h. Each sample was tested using two volumes (5 and 8 μl), and each volume was tested in quadruplicate. Then, the gel was pressed, washed and stained using a Coomassie Blue R 250 stain (Merck, Darmstadt, Germany). An electronic image of the gel was generated using a flatbed scanner (Hewlett Packard—Palo Alto, CA, Supplementary Figure 1), and image analysis with Image-Pro Plus 6.3 Software (Media Cybernetics, Rockville, MD) was used to quantify the stained area of the IHR samples. The stained areas of the extract sample wells were used to calculate the sample concentration in each well using interpolation on the standard curve for the gel. The extract sample concentration was then calculated using a mean estimate calculation and is expressed as Amb a 1 Units/gram (U/g) pollen.

### Assessment of pollen structure

Scanning electron microscopy was performed with air-dried pollen samples that were sputter-coated with gold, and images were obtained in a scanning electron microscope (JSM-6390; JEOL, Peabody, MA). For capturing the images, the software Scanning electron microscopy CONTROLE USER INTERFACE version 8.24 (JEOL, Peabody, MA) was used.

### Induction of allergic disease

To induce experimental allergic disease, we anesthetized 6–8 week old female BALB/c mice with 50 mg/kg ketamine-hydrochloride (Ketanest, Actavis, Italy S.p.A, Nerviano, Italy for Pfizer) and 2 mg/kg xylazine-hydrochloride (Rompun, Bayer AG, Vienna, Austria). Then, we intranasally (i.n.) administered RWP suspended in 50 μl PBS without added adjuvant or PBS alone once a day for 6 doses on days 0, 2, 4, 14, 16, and 18. On day 21, at least 3 mice were assessed for disease parameters, and the rest were left to recover for more than 100 days (termed “recovered” mice). For disease relapse (immunological memory), we instilled a suspension of 10 μg of RWP in 50 μl PBS i.n. to recovered mice and 72 h after the last allergen challenge, and we evaluated disease parameters. RWP were suspended 30 min before i.n. administration of the whole suspension and a fresh suspension was prepared for each administration.

### Airway inflammation

To evaluate airway inflammation, bronchoalveolar lavage (BAL) was carried out *via* tracheostomy. A cannula was inserted into the trachea and then flushed with a total of 1 ml of PBS. The leukocyte cells in the BAL fluid were counted with a hemocytometer (Neubauer chamber). The BAL fluid was cytocentrifuged (Cytospin-4, Shandon Instruments, UK) and then stained with Kwik-Diff (Thermo Fisher Scientific Inc., Pittsburg, PA). Macrophages, eosinophils, neutrophils and lymphocytes were then enumerated based on morphological examination. A total of at least 300 cells per sample were counted.

### Lung inflammation and mucus secretion

Following the BAL, the lungs were resected and perfused with a 4% paraformaldehyde fixative solution. The lungs were placed in histology cassettes and embedded in paraffin. Using a microtome (HM400, Microm, Heidelberg, Germany), sections of 3 μm thickness were prepared and stained with hematoxylin and eosin (H&E) or Periodic-acid-Schiff reagent (PAS). Inflammatory cell infiltration and mucus secretion were assessed with a light microscope (Olympus BX41, Olympus Corp., Tokyo, Japan) and evaluated blindly according to semi-quantitative scoring systems. For lung inflammation, H&E-stained lung sections were graded for the intensity and extent of the inflammatory infiltrates. The histological grade was calculated as the product of the intensity and extent of lung tissue involved. For inflammation intensity: Grade 0: no inflammatory infiltrates; Grade 1: few inflammatory cells around airways/blood vessels; Grade 2: thin layer (<2 cells) of inflammatory infiltrates around airways/blood vessels; Grade 3: thick layer (>2 cells) of inflammatory cells around airways/blood vessels. For the extent of inflammation: Grade 0: no inflammatory infiltrates; Grade 1: inflammatory infiltrates in central airways; Grade 2: inflammatory infiltrates extending to the middle third of lung parenchyma; Grade 3: inflammatory infiltrates spreading to the lung periphery. For the assessment of mucus-production, PAS-stained tissue sections were graded: Grade 0: no mucus-producing cells in airways; Grade 0.5: few mucus-producing cells in the central airways; Grade 1: high mucus production in the central airways; Grade 1.5: sparse mucus-producing cells in the middle airways; Grade 2: abundant mucus production in the middle airways; Grade 2.5: little mucus production in the peripheral airways; Grade 3: many mucus-producing cells in the periphery of the lungs. Representative photomicrographs of stained lung sections were taken with ProgRes®CapturePro 2.9.0.1 software using a Progress Speed XTCore 5 camera (Jenoptik, Jena, Germany) coupled to a light microscope.

### Serum ragweed-specific antibodies

Blood was collected *via* cardiac puncture before the BAL. Sera were acquired after coagulation of the blood samples and centrifugation for 10 min at 15,000 rpm and frozen at −20°C until use. To measure ragweed-specific IgG1 and IgE antibodies, 96-well plates (Nunc Maxisorp, Thermo Fisher Scientific, Roskilde, Denmark) were coated with 5 μg/ml of endotoxin-free ragweed extract dissolved in PBS (Greer Laboratories, Lenoir, NC) and stored overnight at 4°C. On the following day, plates were washed with washing buffer and blocked with 2% bovine serum albumin (BSA) in PBS for 2 h at RT. After washing, serial dilutions of sera were added, and the plates were incubated for ~24 h at 4°C. Plates were washed and incubated with biotinylated anti-IgG1 (Southern Biotech, Birmingham, AL) or anti-IgE (BD Biosciences, San Jose, CA) secondary antibodies for 2 h at 4°C, followed by washing and incubation with streptavidin horseradish peroxidase (HRP, Southern Biotech) for 1 h at RT. After washing, the plates were incubated with 3,3′,5,5′-Tetramethylbenzidine (TMB) substrate solution in the dark for 10 min at RT and the reaction was stopped by the addition of a stop solution (0.18 N H_2_SO_4_). Absorbance was measured with a spectrophotometer at 450 nm.

### Airway hyperresponsiveness

We measured airway hyperresponsiveness (AHR) as a change in airway function at 24 h after RWP rechallenge in response to increasing doses (0.39, 0.78, 1.56, 3.13, 6.25, 12.5 mg/ml) of aerosolized methacholine (Sigma-Aldrich, St. Louis, MI). Anesthetized and tracheostomized mice were restrained and ventilated using Fine Point™ Series Resistance/Compliance equipment (Buxco Electronics Ltd.). Mice were exposed to increasing doses of nebulized methacholine for 3 min with PBS as the baseline. The FinePoint™ (Buxco Electronics Ltd., NY) software was used to calculate parameters of lung function: Airway resistance (RI) and Compliance (Cdyn) of recovered and relapsed mice in response to methacholine expressed as % of PBS baseline.

### Endotoxin measurements

Endotoxin was measured using the Pyrochrome Limulus Amebocyte Lysate (LAL) test (Associates of Cape Cod, Inc., East Falmouth, MA) according to the manufacturer's protocol. Briefly, a 1:10,000 dilution for each pollen was prepared. The samples and the control endotoxin standard (EC010; Associates of Cape Cod, Inc.) (50 μl) were diluted with endotoxin-free water (Associates of Cape Cod, Inc.) and mixed with 50 μl of the LA-Lysate and chromogen substrate for 20 min at 37°C followed by 100 μl of a 50% acetic acid stop reagent. The Optical Density (OD) was recorded at a wavelength of 405 nm.

### Statistics

Data analyses were performed with GraphPad Prism (Version 4.0a, GraphPad Software Inc., La Jolla, CA) applying one-way ANOVA, Student's *t*-test, Mann-Whitney test, and chi-square test. All data are expressed as mean ± SEM, and differences at *p*< *0.05* were considered significant. For AHR, the area under the curve (AUC) was calculated for each experimental animal with resistance (y-axis) vs. the methacholine concentration (mg/ml; x-axis) and analyzed with Student's *t*-test.

## Results

### RWP characterization

We sought to identify differences in allergenicity between RWP samples obtained from distinct geographical locations and environmental conditions (see Supplementary Table 1). We selected cultivated ragweed plant populations from the USA (from Allergon and ALK) and non-cultivated populations collected in Austria. The pollen were from plant populations grown in distinct environments. The AG and ALK populations were cultivated, treated with fertilizers, and grown and harvested in high temperatures with little precipitation. There were differences in the climate between all the cultivated samples. The collected VA1 and VA2 plants were not cultivated. The VA1 plants grew in a rural meadow with some former soil disturbance up to 2 m high, and the VA2 pollen were from short, stubby plants that were close to a heavily used highway and were typically mowed several times a season and were mowed at least once during the season when they were harvested. Neither natural plant population was artificially fertilized or treated with pesticides. Both sites offered relatively nutrient-rich soil at comparable climatic conditions. The details of the climate during the growing season up to the date of harvest were similar without any expected interpretable differences. The AG1 pollen differed from the others because it was the only one defatted with acetone.

**Figure 1:**
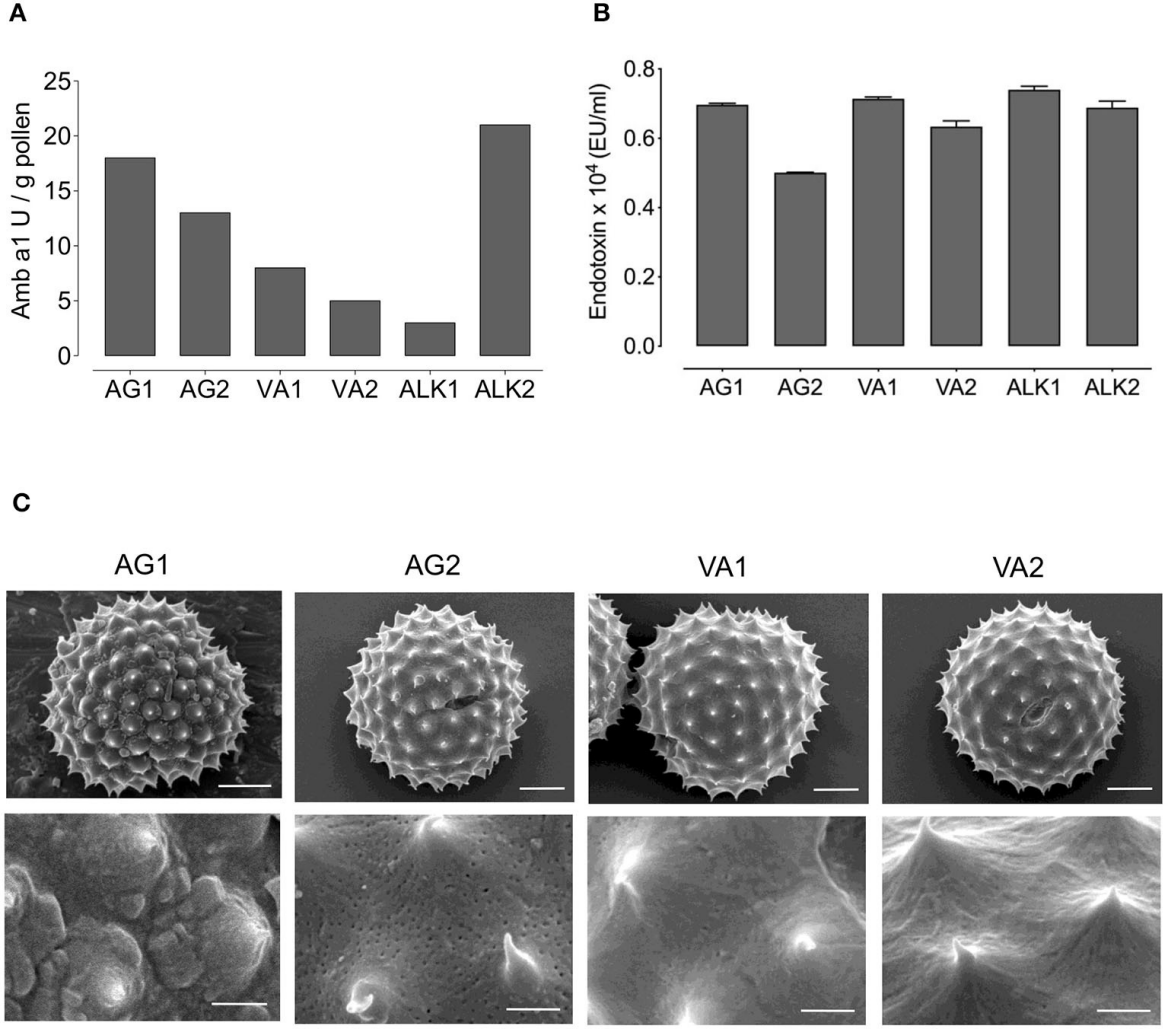
Amb a 1 and endotoxin content, and structure of RWP from different geographical locations. The concentrations of **(A)** Amb a 1 in Units/gram (U/g) and **(B)** Endotoxin in EU/ml in tested RWP samples. **(C)** Scanning electron microscopy photomicrographs were taken of AG1, AG2, VA1, VA2 pollen to examine the structure at magnification of 5,000 × in top row (scale bars = 5 μm) and 21,600 × at bottom row (scale bars = 1 μm).

Based on the differences in the plant populations, we sought to determine whether the pollen structure differed. We examined the structure of RWP using scanning electron microscopy (Figure 1C). The pollen at low power view (5,000 ×) appeared similar with some slight differences within the biological range. Minimal changes in the abortion rate for pollen in sample VA1 compared to the other samples, and some scattered debris in the AG2 sample was observed. We did not detect fungi in the analyzed samples. However, fungal contamination cannot be excluded from samples collected from a natural environment. At high power magnification (21,600 ×), all the pollen samples, except AG2, were similar with intact surface and pollen kit. In contrast, the commercially available defatted AG2 pollen was significantly different because the acetone had removed the pollen kit exposing a porous exine surface.

To further characterize the pollen, we measured the concentration of the major Amb a 1 allergen because it is possible that RWP from plants grown in distinct regions, different seasons, and environmental conditions might differ in the concentration of the allergens and lead to potential changes in allergenicity. Figure 1A illustrates the calculated sample Amb a 1 concentration obtained from the radial immunodiffusion gels (Supplementary Figure 1). Indeed, RWP from plants grown in distinct environments expressed variable amounts of Amb a 1. AG1 and AG2 samples had 18 and 13 U/g of Amb a 1, respectively; Amb a 1 content was 3 U/g in ALK1 and 21 U/g in ALK2 and the samples collected in Austria had 8 and 5 U/g for VA1 and VA2, respectively. These data reveal that the allergen content differed for all samples with AG1, AG2 and ALK2 having the highest and ALK1 and the collected VA1 and VA2 samples having the lowest concentrations.

In addition to allergen concentration, endotoxins adhered to the pollen could also contribute to the immune response by inducing a TLR4-dependent neutrophilic inflammatory response (17). However, in Figure 1B, we show that the endotoxin concentration in all samples was approximately the same, indicating that the relative differences in reactivity to the pollen would not result from endotoxin.

### RWP dose and allergic airway inflammation

**Figure 2:**
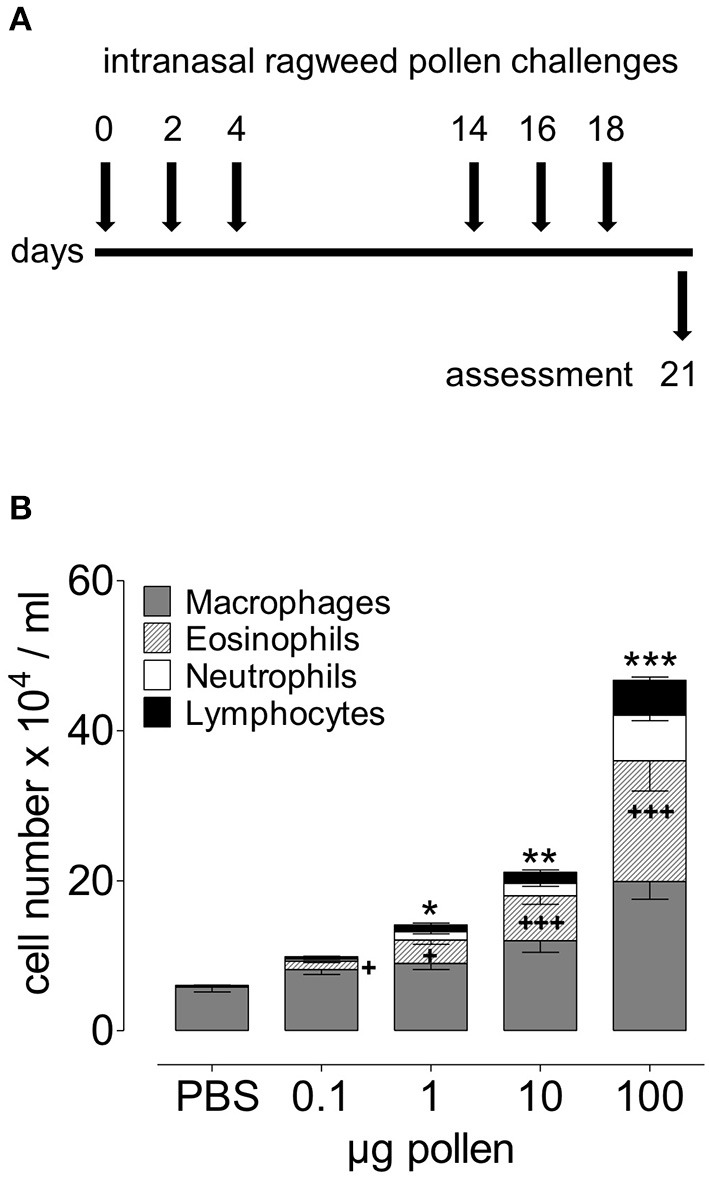
Dose-dependent acute onset of airway inflammation in response to RWP. **(A)** Protocol scheme illustrating administration of PBS alone and increasing doses of AG1 RWP 0.1, 1, 10, 100 μg per 50 μl PBS without added adjuvant over 21 days. **(B)** Total inflammatory cell count and differential counts in BAL 72 h after the last allergen instillation. Data are presented as mean ± SEM and are representative of at least two experiments; *n* = *8*. Asterisks indicate significant differences between PBS and RWP challenged groups, for total cells: *p < 0.05, **p < 0.01, ***p < 0.001, and for eosinophils: ^+^*p* < 0.05, ^+++^*p* < 0.001, one-way ANOVA.

To establish the model of experimental RWP-induced allergic lung inflammation, we selected the untreated RWP AG1 provided by Allergon, which was of high quality and allergenicity with known growth conditions. We administered a dose range of 0.1–100 μg (~30–30,000 pollen grains/dose) RWP suspension into the nose of mice once a day for 6 days over 3 weeks (Figure 2A). Three days after the last pollen instillation, we harvested BAL fluid to assess airway inflammation. We found that mice receiving PBS had 6.06 ± 0.63 x 10^4^ airway inflammatory cells/ml, which contained 95% macrophages, few lymphocytes and almost no eosinophils or neutrophils. Administration of as little as 0.1 μg or ~30 RWP grains boosted airway cell infiltration to 9.89 ± 0.67 ×10^4^ cells/ml with an increase of 11.62 ± 1.5% eosinophils, 2.95 ± 0.5% neutrophils, and 3.38 ± 0.4% lymphocytes. Administered doses of 1 μg (~300 grains), 10 μg (~3,000 grains) and 100 μg (~30,000 grains) induced increasingly higher total airway cell numbers with increases in macrophages, eosinophils, neutrophils and lymphocytes (Figure 2B), illustrating a dose-dependent inflammatory response. The highest dose (100 μg) lead to 7 times more inflammatory cells than in PBS control mice with 31.58 ± 5.1% eosinophils, 13.95 ± 1.7% neutrophils, and 11.03 ± 1.4% lymphocytes. The resulting mixed eosinophil and neutrophil infiltrate demonstrates that RWP induced a severe form of allergic lung inflammation.

### RWP dose and allergic lung inflammation

**Figure 3:**
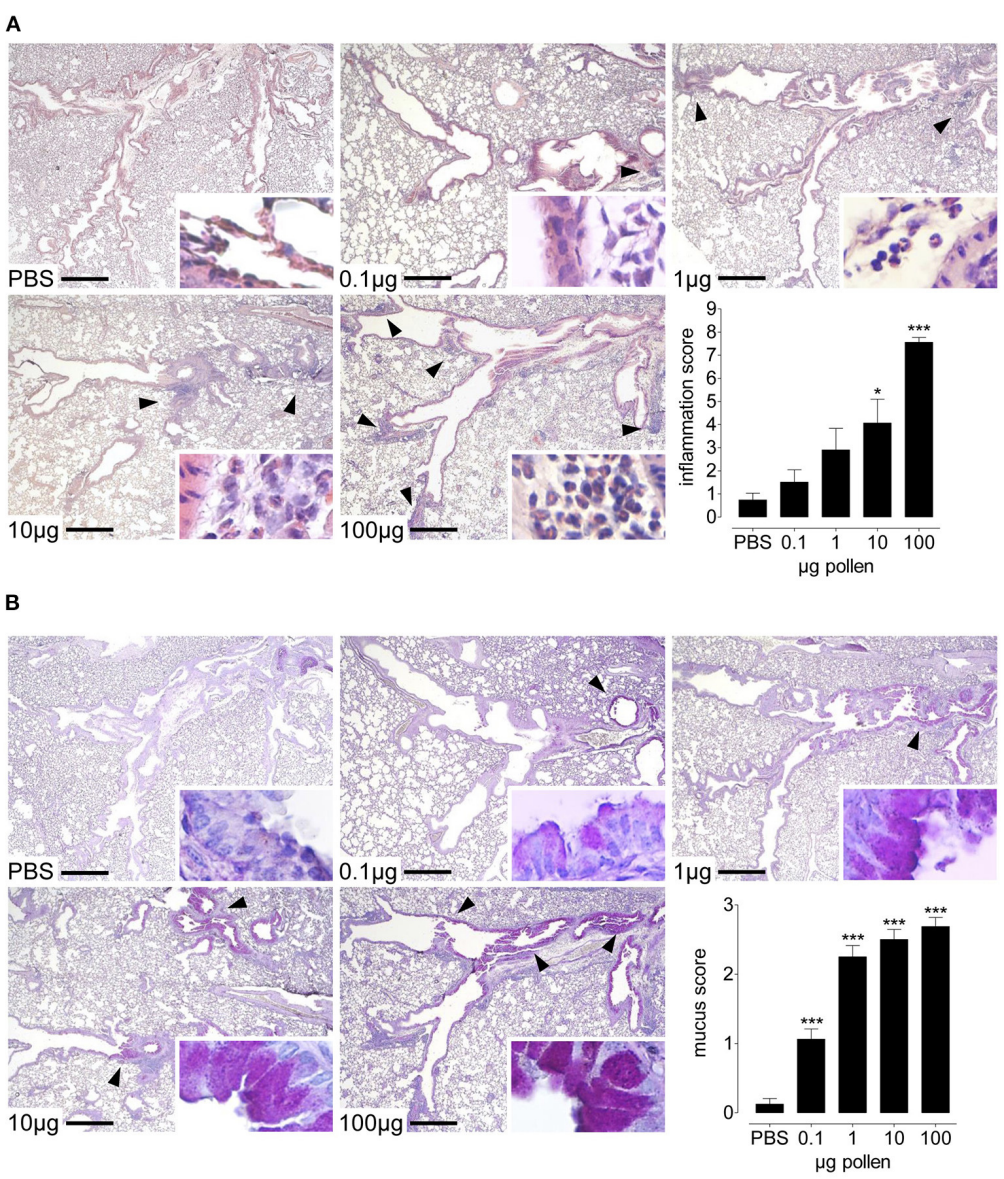
Dose-dependent lung inflammation and mucus production in response to increasing doses of AG1 RWP. Representative photomicrographs of H&E- and PAS- stained lung tissue sections from PBS alone, 0.1, 1, 10, and 100 μg per 50 μl PBS AG1 RWP doses without added adjuvant over 21 days at a magnification of 40 ×. **(A)** In H&E-sections, PBS-treated mice had normal tissue without evidence of inflammation, whereas RWP administration induced inflammatory infiltrates containing eosinophils (insets 400 ×). Arrows indicate inflammatory infiltrates. **(B)** In PAS-stained lung tissue sections, naïve mice had no mucus in the goblet cells but RWP immunized mice had mucus in the goblet producing cells (arrows). Scale bars are 500 μm. Graphs illustrate the quantification of the lung sections using a blinded semi-quantitative scoring system. Data are presented as mean ± SEM and are representative of at least two experiments; *n* = *8*. Asterisks indicate significant differences vs PBS, *p< *0.05*, ***p< *0.001*, chi-square test.

To evaluate the inflammatory infiltrates within the lung tissue, we examined H&E-stained lung tissue sections. Naïve, healthy lungs in untreated mice had no inflammation, while intranasal challenge with RWP induced inflammatory infiltrates (Figure 3A). With the lowest dose (0.1 μg), there were sparse infiltrates predominantly near the central airways with few eosinophils. In contrast, with 1 μg, there were infiltrates in the central and peripheral airways with more infiltrating eosinophils than with the lowest dose. At doses of 10 and 100 μg, there was extensive inflammation throughout the lungs, with many infiltrates in the periphery containing numerous eosinophils. Semi-quantitative scores for the severity and extent of inflammation mirrored the dose-dependent response observed in the airways with an increasing trend that correlated with the dose and statistically significant differences for the two highest doses (Figure 3A).

### RWP dose and mucus hypersecretion

Another feature of allergic asthma is the high production of mucus in the airways. We stained lung sections with PAS to observe the mucus produced in the goblet cells and found that PBS-treated mice have almost no cells producing mucus (Figure 3B). In contrast, mice treated with the lowest pollen dose (0.1 μg) had a few mucus containing cells present in the central airways. At higher doses, we observed more mucus increasingly in the central and peripheral airways, with the highest RWP doses inducing substantially more mucus throughout the lungs. Semi-quantitative scores for the extent of mucus in the lungs was dose-dependent (Figure 3B).

### Lung inflammation in response to diverse RWP

**Figure 4:**
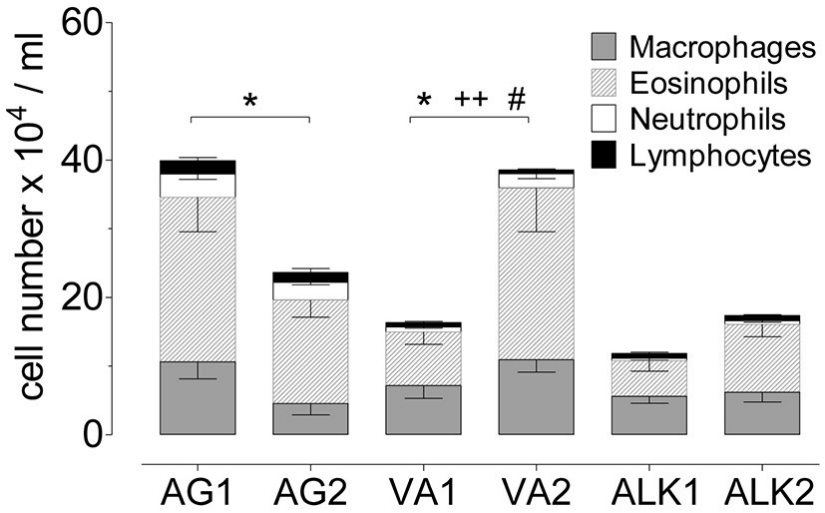
Airway inflammation during the onset of acute allergic disease with diverse RWP from different geographical locations without added adjuvant over 21 days. Total number of cells in BAL with the distribution of macrophages, eosinophils, neutrophils and lymphocytes. Data are presented as mean ± SEM minus PBS control and are representative of at least two experiments; *n* = *8*. Asterisks show significant differences in total cell numbers: *p < 0.05, *t*-test. Significant differences in eosinophils: ^++^*p* < 0.01, and in neutrophils: ^#^*p* < 0.05, Mann-Whitney test.

To determine whether pollen from distinct environments altered the level of sensitization and disease severity, we tested the capacity of each pollen sample to induce allergic lung inflammation. Based on the dose-dependent response in the airways (Figure 2B), we selected the 10 μg dosing schedule. Instilling about 3,000 pollen grains per dose induced a more moderate inflammatory response, and we reasoned that it would be optimal for comparing the pollen samples. We assessed the extent of airway inflammation and found a large range from 11.5 to 40.1 × 10^4^ cells/ml BAL fluid (PBS control subtracted) of infiltrating inflammatory cells in the airways; AG1 (40.1 ± 6.2 × 10^4^ cells/ml) > VA2 (38.5 ± 5.7 × 10^4^ cells/ml) > AG2 (24.9 ± 3.8 × 10^4^ cells/ml) > ALK2 (17.4 ± 3.0 × 10^4^ cells/ml) > VA1 (16.3 ± 3.7 × 10^4^ cells/ml) > ALK1 (11.5 ± 2.7 × 10^4^ cells/ml) (Figure 4). When we evaluated the composition of the airway infiltrates, we found that AG1 and AG2 had a mixed infiltrate consisting of 57–60% eosinophils and 9–11% neutrophils, respectively. In contrast, ALK2 and VA2 had a Th2-type inflammatory infiltrate with between 54 and 60% eosinophils and <4% neutrophils and ALK1 and VA1 had 39–44% eosinophils and <5% neutrophils. RWP collected from different environments qualitatively altered the magnitude and type of allergic response in the airway.

**Figure 5:**
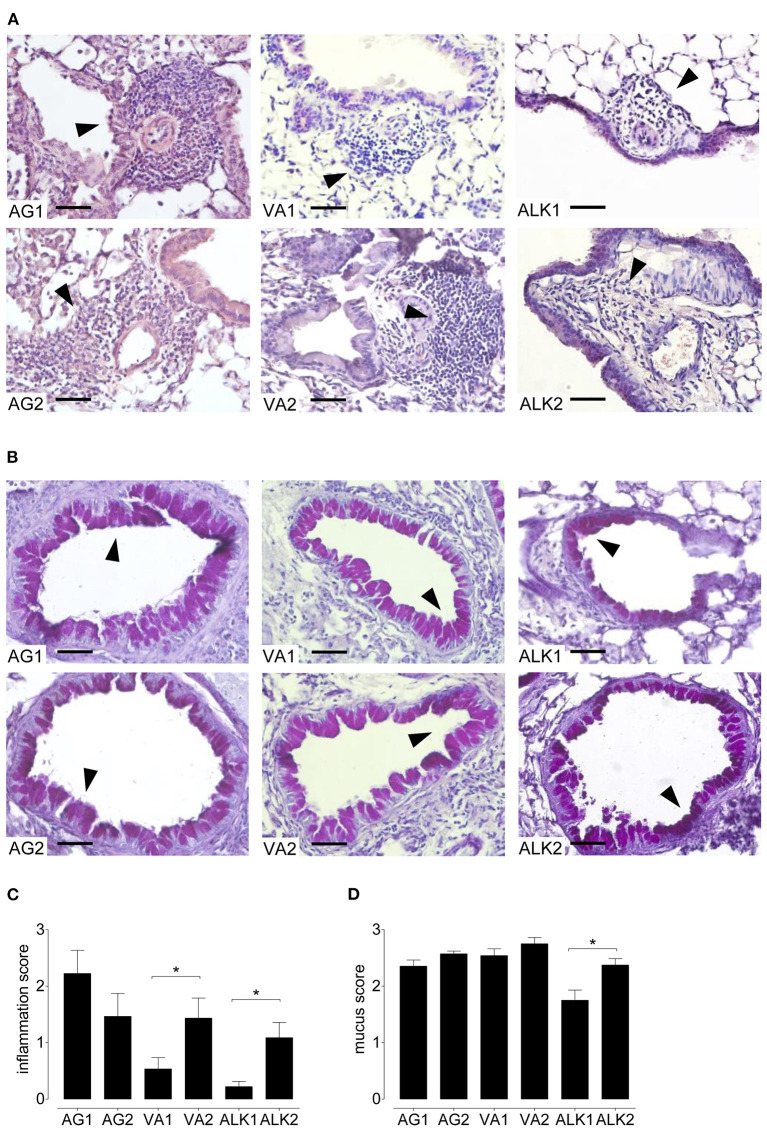
Lung inflammation and mucus secretion during the onset of acute allergic disease with diverse RWP from different geographical locations. Representative photomicrographs of H&E- and PAS- stained lung tissue sections from 10 μg per 50 μl PBS RWP without added adjuvant over 21 days at a magnification of 400 ×. **(A)** In H&E-sections, all RWP samples induced acute inflammatory infiltrates containing eosinophils (arrows), and **(B)** in PAS-stained lung tissue sections, all RWP samples had mucus in the goblet producing cells (arrows). Scale bars are 50 μm. Graphs illustrate the quantification of the lung sections using a blinded semi-quantitative scoring system for lung inflammation **(C)** and mucus production **(D)**. Data are presented as mean ± SEM and are representative of at least two experiments; *n* = *8*. Asterisks indicate significant differences *p < 0.05 in the chi-square test.

Inhalation of each of the RWP samples induced inflammation in the central and peripheral airways (Figure 5A). As in the BAL, mice immunized with AG1, AG2, VA2, and ALK2 had extensive inflammation in the central and peripheral lung parenchyma and contained many eosinophils and some neutrophils. Similar to the airway response, ALK1 and VA1 induced far less lung inflammation with only sparse infiltrates mostly confined to the central airways with few eosinophils. Scores for the severity and extent of inflammation illustrated these differences in a semi-quantitative fashion (Figure 5C).

### Mucus hypersecretion in response to diverse RWP

In lung sections stained with PAS, all RWP induced mucus hypersecretion in central and peripheral airways except for ALK1 pollen, which caused less mucus production and was predominantly in the central airways (Figures 5B,D).

### Immunological “allergic” memory in response to RWP

**Figure 6:**
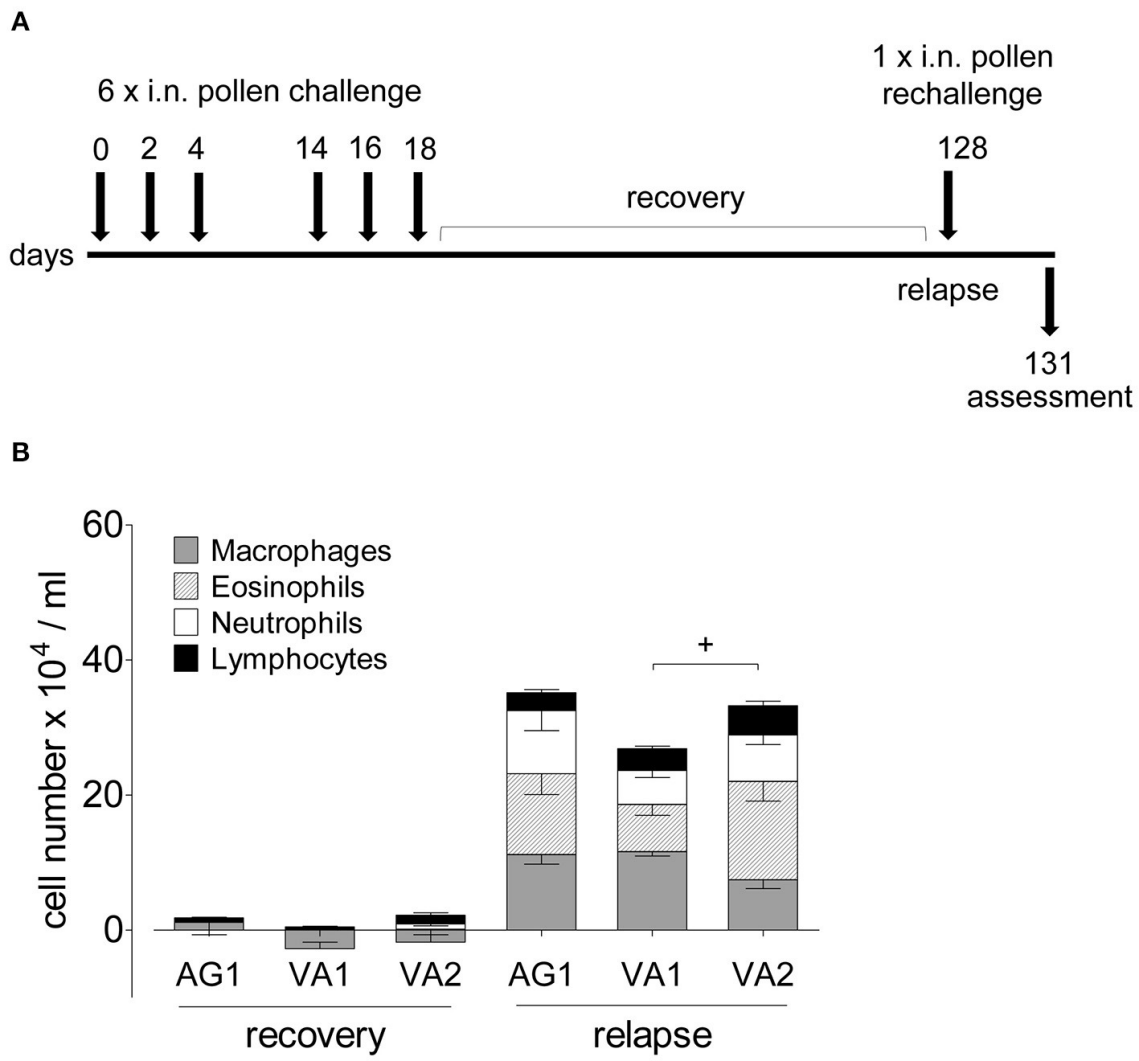
Airway inflammation during disease relapse elicited by AG1, VA1 and VA2 pollen. **(A)** Protocol scheme of disease relapse which illustrates the induction of acute disease with the administration of six RWP doses over 21 days, a recovery period of at least 90 days, followed by a challenge with a single 10 ug intranasal RWP dose before evaluating the mice at 72 h. **(B)** Total and differential BAL cell counts of mice recovered from AG1, VA1, or VA2 pollen-induced disease and at relapse. Data are presented as mean ± SEM minus PBS control and are representative of at least two experiments; *n* = *4–8*. Significant difference in eosinophils: ^+^*p*< *0.05, t*-test.

**Figure 7:**
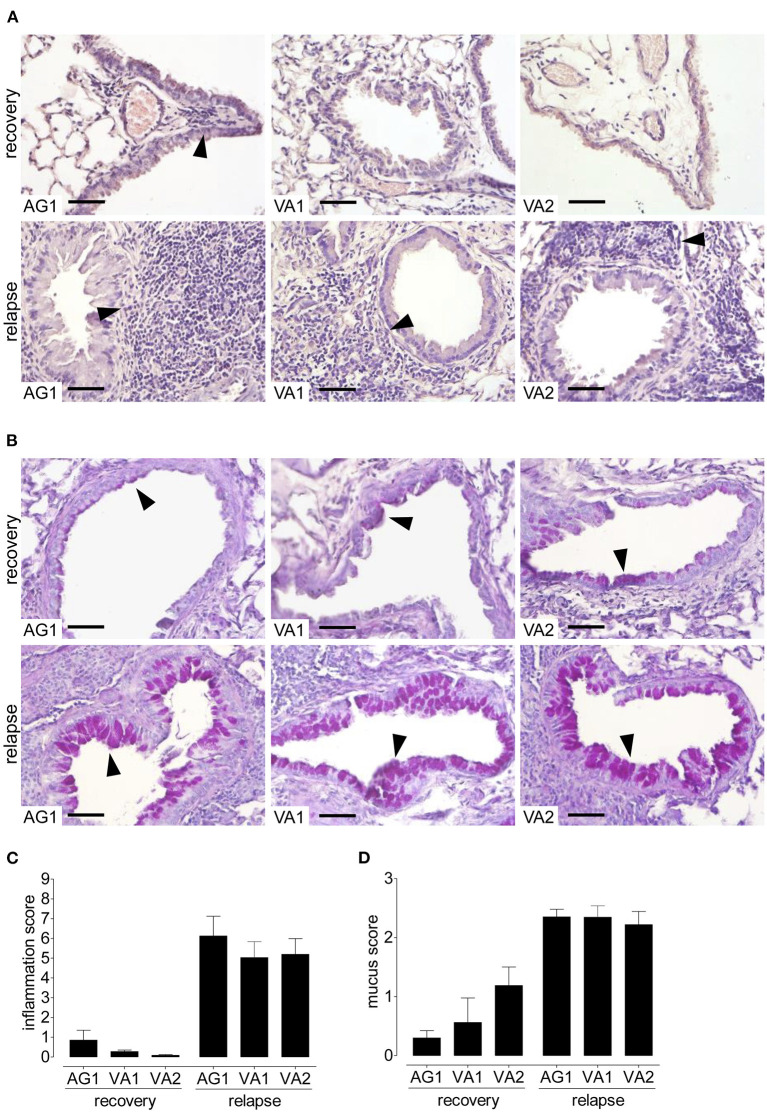
Lung inflammation and mucus secretion during disease relapse elicited by AG1, VA1, and VA2 pollen. Representative photomicrographs of H&E- and PAS- stained lung tissue sections from AG1, VA1, and VA2 pollen (10 μg per 50 μl PBS) at a magnification of 400 ×. **(A)** In H&E-sections, recovered mice have infiltrates without eosinophils, in contrast to relapse with inflammation containing eosinophils (arrows). **(B)** In PAS-stained lung tissue sections, recovered mice have some mucus in goblet cells (arrows) compared with an increase in the rechallenged mice. Graphs illustrate the quantification of the lung sections using a blinded semi-quantitative scoring system for lung inflammation **(C)** and mucus production **(D)**. Scale bars are 50 μm. Data are presented as mean ± SEM and are representative of at least two experiments; *n* = 8.

Because allergic asthma in patients is usually a relapsing-remitting disease caused by a repeated encounter with an allergen, we sought to mimic naturally occurring allergen-induced relapse during the ragweed season. Figure 6A depicts the protocol we used to induce disease relapse. We selected three RWP samples with varying Amb a 1 content (AG1, VA1, and VA2) to investigate allergic memory responses by administering RWP intermittently over 3 weeks to induce acute allergic lung inflammation (Figure 2A). The mice, then recovered from the initial onset of disease for at least 90 days, were once rechallenged intranasally with the same sensitizing RWP (Figure 6A). Recovered mice had mainly macrophages in their airways with few lymphocytes and neutrophils but no eosinophils, illustrating that the mice had recovered from acute allergic airway inflammation. Furthermore, they had small, scattered infiltrates throughout the lung parenchyma containing lymphocytes, macrophages and very few eosinophils (Figure 7A), a pattern that differentiates recovered mice with acute inflammation and naïve, healthy mice. In contrast, RWP rechallenge induced a significant increase in airway inflammation with a mixed eosinophilic and neutrophilic infiltration compared to recovered mice. The relative increase in airway inflammation at relapse was greater than at the initial acute onset of disease illustrating a more robust memory response. RWP similarly increased the total number of airway inflammatory cells at relapse, though, eosinophilia was significantly greater for VA2 than VA1 (Figure 6B) and an intense mixed eosinophilic and neutrophilic inflammatory response in the lung parenchyma which was similar for all the tested samples (Figures 7A,C). Upon close examination of the stained lung sections, there was no evidence of lung remodeling which is similar to our OVA-induced allergic lung inflammation model (18), in which recovered mice maintain quiescent inflammatory infiltrates without remodeling unless they are repeatedly rechallenged (19). Notably, RWP rechallenge also boosted mucus production in airway goblet cells, which added to the persisting mucus observed during recovery from acute disease (Figures 7B,D).

### Systemic immune responses in response to RWP

**Figure 8:**
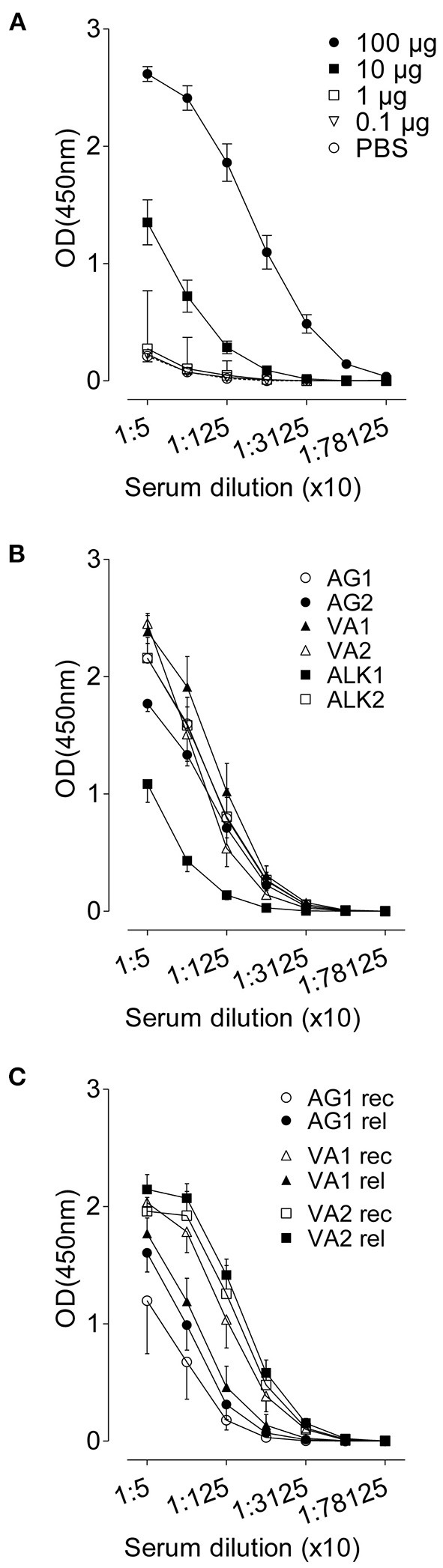
RWP-specific IgG1 serum titres. Allergen-specific antibody responses against **(A)** titrated RWP (AG1) doses during acute onset disease, **(B)** AG1, AG2, ALK1, ALK2, VA1 and VA2 pollen from diverse geographical locations during acute disease onset, and **(C)** AG1, VA1, VA2 pollen during relapse induction. Data are presented as mean ± SEM [minus PBS control for **(B)** and **(C)**] and are representative of at least two experiments; *n* = *4–8*.

A systemic RWP-specific IgG1 antibody response was observed in the sera of mice immunized with 10 and 100 μg of RWP (Figure 8A, Supplementary Figure 2A). However, immunization with 0.1 and 1 μg of RWP did not induce specific IgG1, despite evidence of inflammation and mucus hypersecretion in the lungs (Figures 2B, 3A,B). These data show that a dose equal to and lower than 300 pollen grains could not elicit a systemic antibody response that doses over 3,000 pollen grains were able to induce.

Although allergen-specific IgG1 and IgE in mice are Th2 class antibodies, we observed IgG1 but not IgE even in undiluted sera (data not shown), suggesting that there could be local IgE production but not systemic IgE. However, we could also not detect IgE in the BAL fluid (data not shown). Sera from mice immunized with RWP from different sources had similar titers at the initiation of disease, except for a lower titer in ALK1-immunized samples (Figure 8B, Supplementary Figure 2B), which correlates with a low Amb a 1 content, and other disease parameters, including lung inflammation and mucus secretion.

During recovery, serum IgG1 was highest in mice immunized with VA1 and VA2 and lowest in mice immunized with AG1 (Figure 8C, Supplementary Figure 2C), which does not correlate with Amb a 1 content or inflammation during disease initiation. At disease relapse, IgG1 antibody titers increased in AG1, were almost the same in VA2, and lower in VA1 immunized sera. Several possibilities could explain these differences; one is that the antibody titers were not measured in sera from the same mice pre- and post-allergen exposure and were from different groups of mice, which could lead to variability between groups (Supplementary Figure 2C). Another possibility is that the relapse antibody measurements were done at 72 h after pollen challenge early after relapse and would continue to rise over the following days. Nevertheless, the relative differences between groups did not correlate consistently with Amb a 1 allergen content or disease parameters.

### AHR in response to methacholine during disease relapse

**Figure 9:**
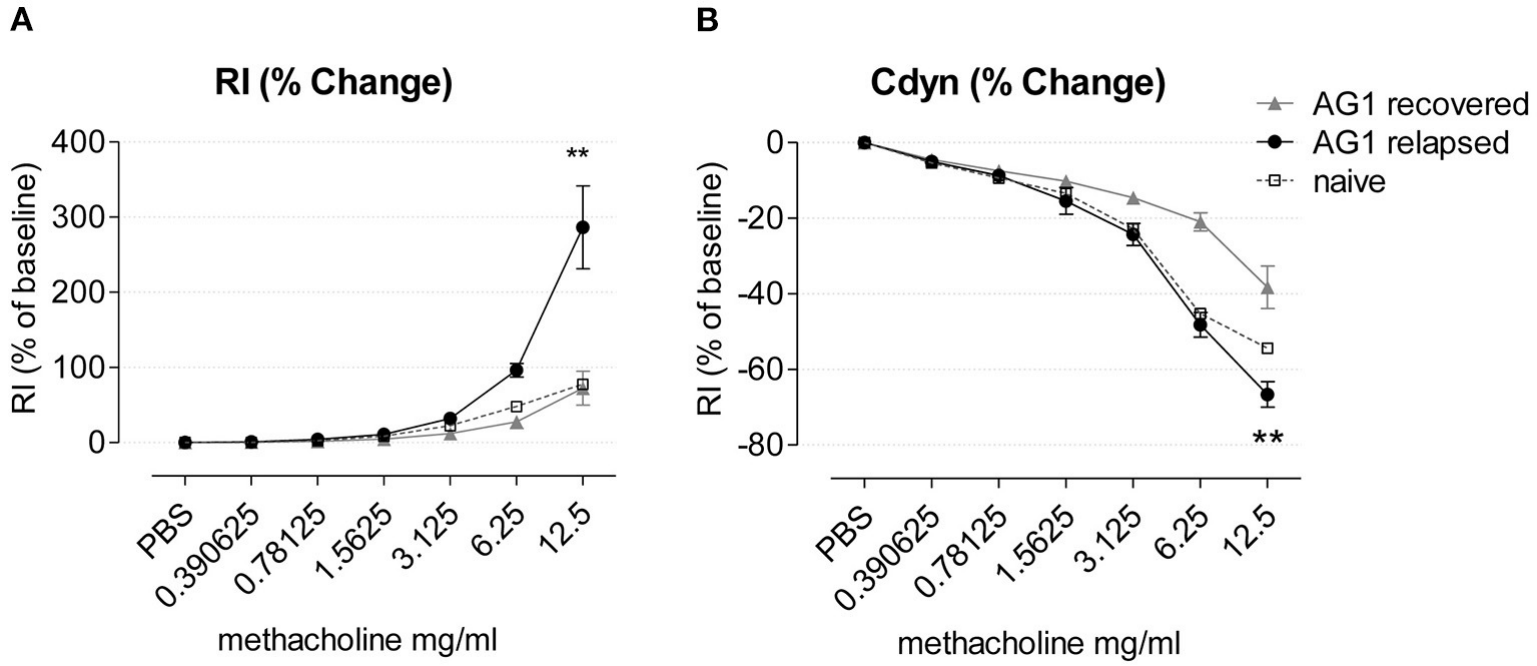
Airway hyperresponsiveness of AG1-immunized mice during recovery and disease relapse 24 h after RWP challenge. **(A)** Airway resistance (RI) and **(B)** Compliance (Cdyn) of mice during recovery and disease relapse (24 h after RWP challenge) in response to methacholine expressed as % of baseline in response to increasing doses of aerosolized methacholine. Data are presented as mean ± SEM and are representative of at least two experiments; *n* = *3–4*. Asterisks indicate a significant difference between recovered and RWP rechallenged groups, **p< *0.01, t*-test of the area under the curve (AUC).

We tested lung function during disease relapse as a particularly important feature of exacerbations in patients with allergic asthma. We found that pollen exposure induced AHR in the presence of increasing doses of methacholine in the RWP-rechallenged compared to the recovered mice, with higher airway resistance (RI) (Figure 9A) and lower dynamic compliance (Cdyn) (Figure 9B). Although, we attempted to measure AHR in mice with acute onset of disease, the mice did not survive the procedure.

## Discussion

Here we show novel RWP-induced mouse models resembling clinically-relevant allergic lung inflammation. Mice administered whole un-manipulated RWP in the absence of added adjuvants develop RWP-specific airway and lung inflammation, mucus hypersecretion, antibodies and allergen-specific immunological memory. A key observation of this study is that the severity of the allergic response to the pollen differed between samples collected in distinct geographical locations as well as on the amount of pollen administered. There was no apparent correlation between disease severity and the concentration of Amb a 1, the major allergen, endotoxin content, or alterations in the structure of the pollen. Our pollen-specific allergic mouse models demonstrate that both the quantity and quality of RWP influence allergic responsiveness and disease severity, suggesting that climate and other environmental factors affect the allergenicity of RWP. We hypothesize that there is an environmental impact on RWP with clinical consequences, which may underlie the increasing sensitization rates and the severity of pollen-induced disease exacerbations, which are likely to worsen in the future.

The natural approach to sensitization underscores the importance of these experimental models of RWP allergic lung inflammation. We instilled whole un-manipulated pollen intranasally in the absence of systemic immunization (e.g., intraperitoneal immunization) or added adjuvants (e.g., alum). Thus, the pollen includes the matrix and potential intrinsic or adhered adjuvants, such as endotoxin, as in a natural environment. Since the first mouse models of allergic lung inflammation in the 1990s, they have become the most frequently used species in allergy research (20–24). Many experimental protocols induce disease with chicken egg ovalbumin (OVA), house dust mite (HDM), purified or recombinant allergens, and often added adjuvant or systemic immunization is required to facilitate disease induction (25–29). Most pollen-induced allergic disease protocols utilize ragweed extract with or without adjuvants (e.g., alum) with systemic sensitization (i.p.) followed by a respiratory challenge (30–34) or with high RWP doses (e.g., 1 mg/dose i.n.) and mainly with commercial ragweed samples without comparing different lots (35–38). We focused on the natural sensitization process occurring in the human respiratory tract. We developed clinically relevant models of allergic airway disease to study the allergenic potential of RWP from different locations and environments.

Using our experimental model, we addressed the dose of RWP necessary for sensitization. We found that 0.1 μg (~30 pollen grains) of RWP instilled i.n., six times intermittently within 3 weeks for a total of ~180 pollen grains was sufficient to sensitize mice and induce mild inflammation in the airways and mucus secretion. Our data illustrate that the dose necessary for sensitization in the mice is low and imply that only a few pollen grains could sensitize individuals. However, it is very difficult to compare the doses necessary to elicit a response in mice in an experimental setting with the concentration humans are exposed to over a whole pollen season, where clinical findings show that a threshold of >5,000 RWP grains/ m^3^ /year is necessary for sensitizing patients (14). In an often-cited study, the number of RWP grains required to elicit hay fever symptoms was calculated (39). The authors applied RWP directly to the nasal mucosa and found that 20,000 to 30,000 grains instilled into the nose produced symptoms and then they estimated that environmental ragweed-sensitive patients will have symptoms with a concentration of 25 pollen granules/yard^3^ based on a person inhaling 20 cubic yards of air in 24 h resulting in a total of 500 pollen grains inhaled/day with the caveat that not all would land on the mucous membranes. Typically, RWP sensitization occurs after 3–5 RW seasons and 5,000 pollen grains/m^3^/year, an individual would need a total of ~9,000–25,000 pollen grains/m^3^ for sensitization in 3–5 years, respectively. These observations illustrate the capacity of RWP to cause disease in mice at low concentrations compared to the dose needed for humans.

We also found that increasingly higher doses of pollen correlated with the mice developing more intense inflammation. When we increased the dose to 1 μg (~300 pollen grains), 10 μg (~3,000 pollen grains) and 100 μg (~30,000 pollen grains), the number of airway inflammatory cells increased, most notably the percentage of eosinophils and neutrophils, with 100 μg leading to the most severe disease. Thus, titrated doses of RWP caused increasing severity of acute airway and lung disease, as previously shown in mice and patients (10, 11, 13, 14, 40, 41). Because endotoxin is present in the samples, increasing pollen dose also increases the endotoxin content and together they could induce the more intense, mixed inflammatory response that we observed. However, endotoxin is ubiquitous in a natural environment and its content was similar between samples, it is likely that it contributes equally to the immune response against the tested pollen.

In addition to local lung responses, we also evaluated the systemic reaction to inhaled RWP by measuring serum allergen-specific antibodies. Surprisingly, we detected IgG1 antibodies in mouse sera upon administration of 10 and 100 μg RWP, but not with of 0.1 and 1 μg. These results suggest that the lower doses of ~300 pollen grains could induce local lung inflammation and mucus hypersecretion without allergen-specific IgG1. It is possible that the ELISA used in our experiments was unable to detect low IgG1 titers with low RWP doses using our ELISA or that after the last allergen challenge, there was a delay that could enable us to detect IgG1 at a later time.

More puzzling is that we could not detect allergen-specific IgE in any ragweed-immunized mice, which contradicts other ragweed models in which both serum allergen-specific IgG1 and IgE were detected (37, 42, 43). However, these models were done with substantially different protocols in which RWP with alum were administered by intraperitoneal injection (37), or RWP extract were instilled intranasally (42) or RWP were administered intranasal over 5 weeks followed by an intranasal challenge with Amb a 1 (43). Our results, however, support previous findings in which immunized animals developed allergic lung inflammation without the IgE (29, 38, 44). Another possibility is that the IgG1 titers are so high that they mask IgE. To address this issue is straightforward with single protein allergens, like ovalbumin. Antigen-specific IgE can be unmasked by excluding IgG1 with an ELISA in which the plate is coated with anti-IgE, followed by the addition of biotinylated antigen and then a conjugated streptavidin. However, this approach is problematic for ragweed because the pollen are a complex mixture of allergens, making the biotinylation step more complicated. Notably, it is also possible that our protocol induced IgE-independent disease, as previously reported (19, 45).

After establishing the dose-dependent effect on disease, we selected a moderate dose of 10 μg (~3,000 pollen grains) for testing differences between RWP from diverse environments. At the same RWP doses, we observed significant differences in the ability of the pollen samples to induce allergic disease. We found that exposure to AG2 and AG1 induced extensive mixed eosinophilic and neutrophilic inflammatory cell infiltration in the airway and lungs, which is remarkably similar to inflammation in patients with severe asthma (27, 46, 47). In contrast, VA2 and ALK2 induced a predominantly Th2 eosinophilic inflammatory response, whereas VA1 and ALK1 were both less aggressive and induced mild inflammation compared to AG1 and AG2. ALK1 also induced the lowest allergen-specific IgG1 titer. These data demonstrate that RWP at the same dose but originating from distinct sources or locations markedly altered disease severity and the composition of inflammatory cells in the airways, suggesting that along with the quantity, there are additional pollen properties that can make the initiation of disease worse.

To further identify potential properties of the pollen samples that influenced disease induction, we measured the content of the major allergen Amb a 1, a pectate lyase recognized by over 90% of ragweed-sensitized patients and induces high antibody levels in mice and humans (48–50). We found that the Amb a 1 content ranged from 3 to 21 U/g depending on the sample and found no apparent correlation between allergen concentration and disease severity. AG1 pollen induced the most severe disease and had a high Amb a 1 content while the acetone-treated AG2 pollen samples had lower levels of Amb a 1 and induced less severe disease. In contrast, ALK2 had high Amb a 1, but did not cause as severe disease as AG1. The VA2 pollen were also highly allergenic but had a low Amb a 1 content, suggesting that despite being the immunodominant allergen in RWP, Amb a 1 content does not appear to influence disease severity. This is supported by studies showing that i.n. administration of Amb a 1 alone does not cause airway inflammation or increase specific IgG1 (32, 44). However, we found significantly higher scores of tissue inflammation as well as mucus production and RWP-specific IgG1 with higher Amb a 1 levels in ALK2 compared to lower levels in ALK1, suggesting that there is a partial impact of Amb a 1 content.

The differences in Amb a 1 content, even though Amb a 1 does not correlate with disease severity could be attributed to a multitude of environmental factors like location, weather, timing of sampling and changes occurring during the storage or processing, e.g., defatting which reduced Amb a 1 content and may be related to acetone disruption of the pollen membranes. Indeed, the AG2 pollen caused less intense inflammation but a similar mixed eosinophilic, neutrophilic pattern compared to untreated pollen, suggesting that other allergenic components may be partially removed from the pollen upon acetone treatment, but it is likely that the samples differed because they were from different seasons and areas.

Environmental factors might also underlie our observed disease-enhancement with specific RWP samples. A myriad of factors could explain the differences including weather conditions during the growing season, e.g., amount of rain, temperature etc., soil properties, air pollution, use of fertilizers or pesticides, repeated mowing or other mitigation approaches, the timing of the harvest, time since collection, conditions for pollen storage after collection, e.g., temperature and humidity. For example, the VA2 pollen collected from the highway roadside might induce more severe disease because of the repeated mowing of the plants due to roadside maintenance or heavy highway pollution. Pollution has been a factor previously reported to alter pollen (51). Lectins, and other contaminating air and ground pollutants (e.g., diesel fuel particles, NH_4_NO_3_), or other particulate matter on the pollen could potentially influence its allergenicity. Carbon dioxide (CO_2_) increases the content of the major allergen Amb a 1 (52–54), and NO_2_ up-regulates Amb a 1 encoding transcript levels (55), increases Amb a 1 isoforms and other allergens, with enhanced overall nitrosylation and increased Amb a 1 allergenicity (56). Furthermore, the increasing number of ragweed allergic patients appears to correlate to high airborne pollen concentration, which is associated with climate change, i.e., elevated temperatures and CO_2_ levels (3–6). The impact of environmental factors was observed with allergenic birch pollen, with ozone, increased temperatures, and pollution enhancing the allergenicity by Bet v 1-nitration (57–60). Additionally, urban birch pollen samples differed in protein expression and chemotactic activity on human neutrophils (61), grass pollen from plants exposed to cadmium, ozone, or an urban environment had higher allergenic potential, and increased *Cupressaceae* pollen from polluted areas had higher allergen content (62–66). Although, it is tempting to speculate that differences between the effects of VA1 and VA2 pollen are due to pollution, testing of more replicates of rural or urban area pollen sources would be needed. Furthermore, controlling the environment during the growing of the ragweed plants will be essential for determining the conditions that alter the pollen and subsequently influence their allergenicity.

Other potential environmental factors including contamination with endotoxin and fungi could potentially explain our observations. However, we did not detect fungal or spore contamination and the endotoxin content was similar for all pollen samples, suggesting that these factors do not underlie the differences between the pollen. Taken together, our findings and previous reports suggest that changes in the environment where ragweed plants grow or where their pollen are released and transported may alter them in a way that could increase the prevalence and severity of disease onset.

Allergic asthma in patients is a relapsing-remitting disease with allergen inducing disease exacerbations. To determine whether RWP-immunized animals also developed disease relapse and to test the magnitude and character of the response to different pollen samples, we elicited an allergen-induced relapse based on an immunological 'allergic' memory model with purified OVA protein (18, 67). Disease relapse followed acute disease onset and a recovery period of at least 90 days before rechallenging the mice with one i.n. dose of 3 selected RWP samples. We found that the secondary challenge induced a significant inflammatory response in the airways and lungs for the tested pollen. The disease relapse was severe for each pollen sample irrespective of the initial onset of disease or the Amb a 1 content. AG1, VA1, and VA2 significantly increased the numbers of eosinophils and neutrophils, demonstrating that the disease relapse was more severe than the acute disease. Furthermore, the VA2 pollen caused even more inflammation than VA1 with significantly higher airway eosinophil counts, similar to acute onset disease. RWP induced AHR in response to methacholine compared to recovered mice illustrating the full spectrum of memory responses in the model. AHR was also tested in mice with acute onset of disease, but the mice did not survive the procedure, implying a remarkable, unexplained sensitivity to methacholine during disease initiation.

RWP-induced disease relapse mimics disease exacerbations in patients. But can the RWP dose for eliciting a response in humans compare with mice? Using the findings of Feinberg and Steinberg again, we note that 20,000–30,000 RWP grains elicited symptoms in humans, while it only takes 3,000 RWP grains to induce a robust relapse in mice. There are possible explanations for disparate eliciting doses between mouse and patient. Firstly, there are expected differences of the inhaled RWP grain deposition in the lung based on the total fraction of inhaled aerosol that deposits in the respiratory tract including the nose and mouth, the respiratory tract deposited particle dose rate, which is expressed as the concentration of particles in the inhaled air by the minute ventilation which equals the deposition in the respiratory tract over time and depends on the morphology of the lungs and respiratory parameters including respiratory rate and volume. Secondly, disease readouts differ. In the experiment with humans, the values were for eliciting hay fever symptoms, not the allergic lung inflammation, mucus hypersecretion and AHR we tested in the mice. Thirdly, differences could be attributed to the timing of symptoms. There is a significant lag of 3–18 days after the first day of pollen exposure for the development of ocular, nose and lung symptoms (68). Despite differences in the RWP quantity required to elicit a relapse, RWP-induced disease relapse in mice mimics disease exacerbations in patients and provides a valuable model for further study of diverse RWP and potential therapeutic intervention.

In summary, our findings illustrate that inflammatory cell recruitment is dependent on the quantity and quality of RWP and differs greatly depending on environmental factors of distinct geographical regions affecting the RWP, which could lead to different sensitization rates and disease exacerbation severity. Further investigation is necessary to elucidate the contribution of pollen characteristics (e.g., proteomics, metabolomics) and the underlying mechanisms.

## Data availability statement

The raw data supporting the conclusions of this article will be made available by the authors, without undue reservation.

## Ethics statement

This study was carried out in strict accordance with the guidelines for the care and use of laboratory animals of the Austrian Ministry of Science. The protocol was approved by the Committee on the Ethics of the Austrian Ministry of Science (Number: GZ: 66.009/0330-II/3b/2013). All painful procedures were performed under anesthesia, and all efforts were made to minimize suffering.

## Author contributions

S-HL designed and performed experiments, analyzed the samples and contributed to the manuscript preparation. SK performed experiments and contributed to the manuscript preparation. GK assisted in the collection of RWP. AB acquired the scanning electron microscopy photomicrographs and evaluated them. WW provided knowledge on pollen characteristics. JD performed Amb a 1 measurements. OH was responsible for the endotoxin assay. ME supervised experiments, analyzed the data, and contributed to the manuscript preparation. All authors read and approved the final version of the manuscript.

## Funding

This project was supported by the European Community Framework Programme 7, ATOPICA (Atopic diseases in changing climate, land use and air quality), grant agreement no. 282687.

## Conflict of interest

The authors declare that the research was conducted in the absence of any commercial or financial relationships that could be construed as a potential conflict of interest.

## Publisher's note

All claims expressed in this article are solely those of the authors and do not necessarily represent those of their affiliated organizations, or those of the publisher, the editors and the reviewers. Any product that may be evaluated in this article, or claim that may be made by its manufacturer, is not guaranteed or endorsed by the publisher.

## Abbreviations

AHR: airway hyperresponsiveness
AUC: area under the curve
BAL: bronchoalveolar lavage
BSA: bovine serum albumin
Cdyn: dynamic compliance
CO_2_: carbon dioxide
H_2_SO_4_: sulphuric acid
H&E: hematoxylin and eosin
HDM: house dust mite
HRP: horseradish peroxidase
i.n.: intranasal
i.p.: intraperitoneal
NH_4_NO_3_: ammonium nitrate
NO_2_: nitrogen dioxide
OVA: chicken egg ovalbumin
PBS: phosphate-buffered saline
PAS: periodic-acid-Schiff
RI: airway resistance
RT: room temperature
RW: ragweed
RWE: ragweed extract
RWP: ragweed pollen
SEM: standard error of the mean
TMB, 3, 3′, 5: 5′-Tetramethylbenzidine.
