# Supplementary material for: Influence of the environment on ragweed pollen and their sensitizing capacity in a mouse model of allergic lung inflammation
Source: Front Allergy. 2022 Aug 5;3:854038. doi: 10.3389/falgy.2022.854038 (PMC9390857; doi:10.3389/falgy.2022.854038)
Supplement: Supplementary file 1 [file Presentation_1.pdf]

Supplementary Material

1 Supplementary Figures and Tables

1.1 Supplementary Figures

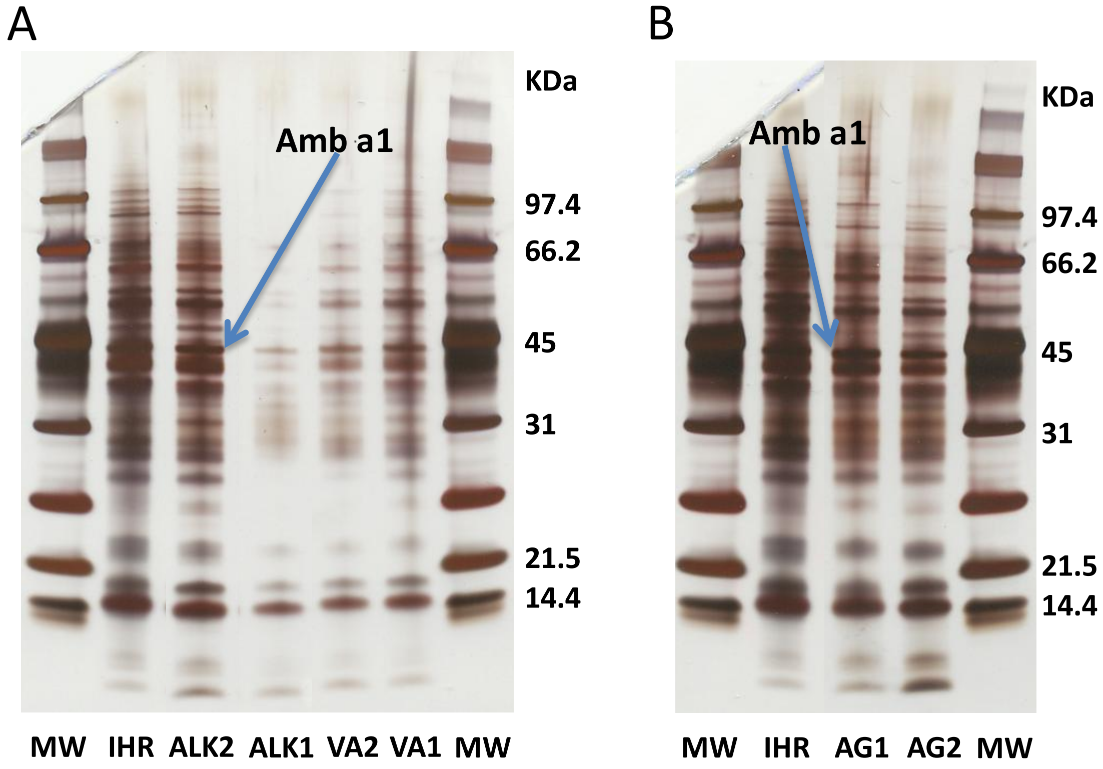

Supplementary Figure 1. Radial immunodiffusion gels of different pollen samples. An ALK in-house reference sample (IHR), molecular weight marker (MW) and the RWP extract samples are shown. Image analysis was used to quantify the stained area of the IHR samples. The stained areas of the RWP extract samples were used to calculate the sample concentration in each well using interpolation on the standard curve for the gel. The RWP extract sample concentration was then calculated using a mean estimate calculation and is expressed as Amb a 1 Units/gram (U/g) pollen.

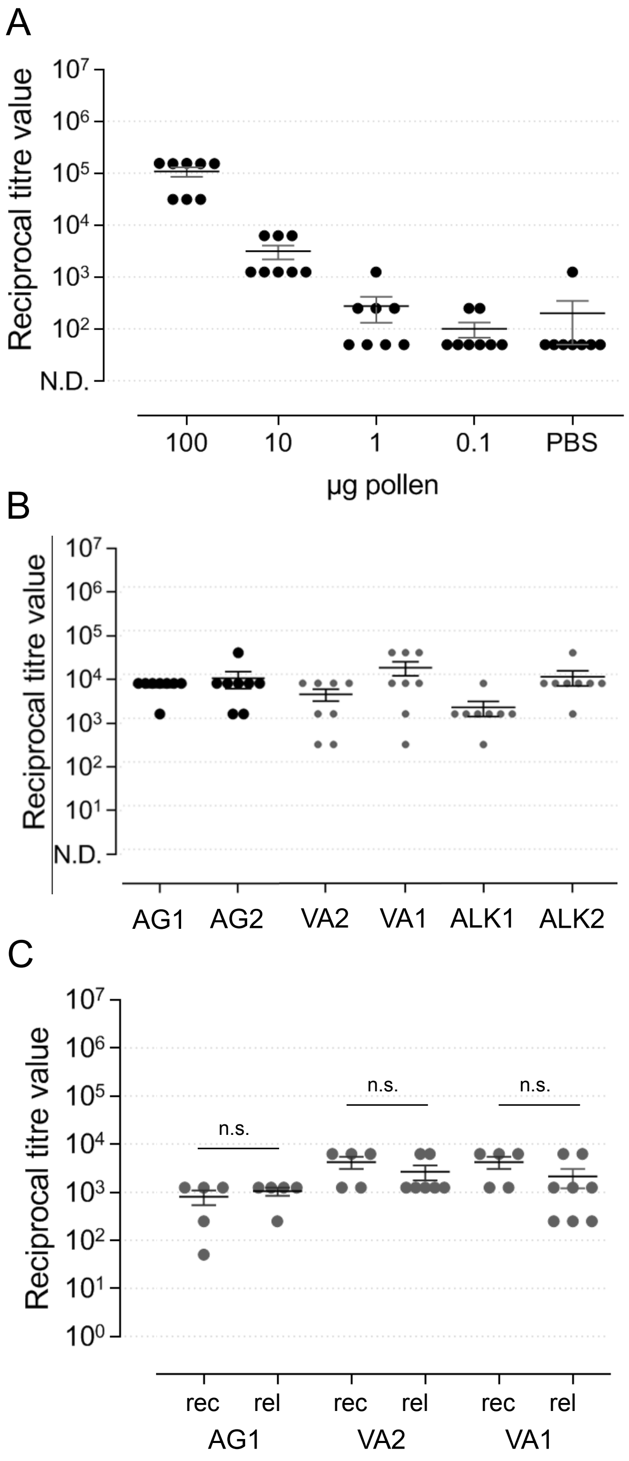

Supplementary Figure 2. Ragweed pollen-specific IgG1 titres of sera harvested 3 days after the last i.n. challenge, assessed via standard ELISA. (A) Acute asthma model with different AG1 pollen doses. (B) Acute asthma model with pollen from different sources. (C) Memory asthma model with AG1 and collected VA1, VA2 pollen after recovery (rec) and at relapse (rel). Data are presented as mean ± SEM and are representative of at least two experiments; n = 4 - 8.

1.2 Supplementary Tables

Sample	Location	Year	Growing Conditions	Harvest conditions	Storage	Analysis
AG1	Jasper County, Missouri, USA	2011	• Cultivated field, no-till drill wheat and broadcast ragweed seed 5/11/2010<br>• Fertilized nitrogen, phosphorus, and potassium on 24/11/2010 and 15/3/2011<br>• No pesticides used<br>• Wheat cut 7/3/2011<br>• Drought conditions with high heat 35 -40.6ºC<br>• Rain on 8/10-11/2011 and 9/4/2011<br>• Ragweed grew naturally 1-1.3 m tall<br>• Blooming period 9/2-13/2011<br>• For weather conditions: *http://agebb.missouri.edu/weather/realtime/lamar.asp*	• Daytime temperatures cooled to 27-34ºC during collection<br>• Sifted by screen and wind<br>• Vacuum dried at <33ºC for 24h<br>• Stored at +2-8ºC<br>• Shipped at ambient temperature<br>• Stored in aluminium containers	• Defatted with acetone for commercial use<br>• Stored -20ºC	• 94.93% purity<br>• Mold spores, foreign pollen, non-plant particles <1<br>• Moisture content <7<br>• Amb a 1 content<br>• Endotoxin content<br>• Ultrastructure
AG2	Barton County, Missouri, USA	2012	• Wild field after cultivated wheat<br>• Natural growth after wheat harvest 1-1.3 m tall<br>• Summer dry condition<br>• Blooming period 3-14/9/2012 and on 20-24/9/2012 on two separate fields<br>• For weather conditions: *http://agebb.missouri.edu/weather/realtime/lamar.asp*	• Rained twice during collection causing high humidity<br>• Sifted by screen and wind<br>• Vacuum dried at <33ºC for 24h<br>• Stored at +2-8˚C<br>• Shipped at ambient temperature<br>• Stored in aluminium containers	• Untreated<br>• Stored -20˚C	• 97.16% purity<br>• Mold spores, foreign pollen, non-plant particles <1<br>• Moisture content <7<br>• Amb a 1 content<br>• Endotoxin content<br>• Ultrastructure
ALK1	Mason County, Havana, Illinois, USA	2007	• Collected from plants cultivated in a wheat and ragweed cropping system<br>• Fertilized two times per season with nitrogen, phosphorus, and potassium<br>• No pesticides<br>• Soil type is a fine sandy loam with a high drainage level<br>• Temperatures were 16ºC to 30ºC from May to September<br>• Ragweed grows to a height of 1.0-1.5 m<br>• Average temperatures and precipitation	• Collection started in early September<br>• Pollen collected using a tractor-mounted vacuum system- intake funnels at 1m<br>• Dried using a custom drying chamber<br>• Sieved using a rotating screen sieve<br>• Frozen on-site<br>• Stored at -20˚C<br>• Transported at -20˚C<br>• Stored in HDPE containers	• Untreated<br>• Stored -20˚C	• >94% Pollen purity<br>• No evidence of mold spores or fungi<br>• plant parts <5%<br>• foreign material <1%<br>• other pollen <1%<br>• Moisture <7%<br>• Amb a 1 content<br>• Endotoxin content

**ALK2**	Mason County, Havana, Illinois, USA	2012	• Collected from plants cultivated in a wheat and ragweed cropping system<br>• Fertilized two times per season with nitrogen, phosphorus and potassium<br>• No pesticides<br>• Soil type is a fine sandy loam with a high drainage level<br>• Temperatures were 16°C to 30°C from May to September<br>• Ragweed grows to a height of 1.0-1.5 m<br>• Extreme drought year with almost no precipitation	• Collection started in early September<br>• Pollen collected using a tractor-mounted vacuum system- intake funnels at 1m<br>• Dried using a custom drying chamber<br>• Sieved using a rotating screen sieve<br>• Frozen on-site<br>• Stored at -20°C<br>• Transported at -20°C<br>• Stored in HDPE containers	• Untreated<br>• Stored -20°C	• >94% Pollen purity<br>• No evidence of mold spores or fungi<br>• plant parts <5%<br>• foreign material <1%<br>• other pollen <1%<br>• Moisture <7%<br>• Amb a 1 content<br>• Endotoxin content
**VA1**	Bruckneudorf, Austria 48°00'24.4"N 16°45'38.2"E	2013	• A large undisturbed Ambrosia population<br>• Plants were grown in an uncultivated meadow with some soil disturbance<br>• The plants did not suffer from any human influence<br>• The soil was calcareous cernozema with a high nutrient supply and was not artificially fertilized but had enough nitrogen for nice growth<br>• 1-2 m tall fully grown<br>• Altitude: 164 m	• The collection was in August<br>• According to the Wiener Neustadt, airport weather station, the mean monthly temperatures in 2013 for the growing season (°C): March: 3.0; April: 11.1; May: 15.1; June: 18.1: July: 22.3; August. 21.1<br>• The sum of precipitation per month (mm): March: 37, April: 9 (very dry), May: 88, June: 110, July: 4 (dry), August: 75;<br>• 1-2 m tall plants including roots removed from the soil and put in water containers in a glasshouse<br>• Pollen collected from the area underneath and around the vases	• Untreated<br>• Dried, sieved<br>• Stored -20°C	• No evidence of mold spores or fungi<br>• Amb a 1 content<br>• Endotoxin content<br>• Ultrastructure
**VA2**	Vienna, Austria highway A22 48°16'38.9"N 16°22'02.9"E	2013	• Ambrosia plants grew on a highway road embankment<br>• The soil was calcareous artificial soil with medium to high nutrient supply and was not artificially fertilized but had enough nitrogen for nice growth<br>• Heavy traffic pollution exposure but there were no specific data about the emissions<br>• Small size due to regular mowing as part of roadside maintenance- this population had at least 1 cutting before transplantation of the plants<br>• Altitude: 161 m	• The collection was in August<br>• According to the Vienna, Hohe Warte weather station, the mean monthly temperatures in 2013 for the growing season (°C): March: 3.0; April: 11.7; May: 15.0; June: 18.7: July: 22.9; August. 21.3<br>• The sum of precipitation per month (mm): March: 39, April: 13 (dry), May: 136, June: 144, July: 11 (dry), August: 58<br>• 20-30 cm tall plants including roots removed from the soil and put in water containers in a glasshouse<br>• Pollen were collected from the area underneath and around the vases	• Untreated<br>• Dried, sieved<br>• Stored -20°C	• No evidence of mold spores or fungi<br>• Amb a 1 content<br>• Endotoxin content<br>• Ultrastructure

Supplementary Table 1. RWP sample characterization.
